# Developmental Programming and Postnatal Modulations of Muscle Development in Ruminants

**DOI:** 10.3390/biology14080929

**Published:** 2025-07-24

**Authors:** Kiersten Gundersen, Muhammad Anas

**Affiliations:** 1 Department of Animal Sciences, North Dakota State University, Fargo, ND 58105, USA; kiersten.gundersen@ndsu.edu (K.G.);; 2 Project Planning and Development Unit, Livestock Wing, Ministry of National Food Security and Research, Islamabad 44000, Pakistan

**Keywords:** myogenesis, maternal nutrition, satellite cells, myogenic regulatory factors, postnatal hypertrophy, prenatal programming, carcass quality, myostatin, IGF-1, meat production

## Abstract

Prenatal and postnatal skeletal muscle development in ruminants is coordinated by interactions between genetic, nutritional, epigenetic, and endocrine factors. This review focuses on the influence of maternal nutrition during gestation on fetal myogenesis, satellite cell dynamics, and myogenic regulatory factors expression, including *MYF5*, *MYOD1*, and *MYOG*. Studies in sheep and cattle indicate that nutrient restriction or overnutrition alters muscle fiber number, the cross-sectional area, and the transcriptional regulation of myogenic genes in offspring. Postnatally, muscle hypertrophy is primarily mediated by satellite cells, which are activated via *PAX7*, *MYOD*, and *MYF5*, and regulated through mechanisms such as CARM1-induced chromatin remodeling and miR-31-mediated mRNA expression. Hormonal signaling via the GH–IGF1 axis and thyroid hormones further modulate satellite cell proliferation and protein accretion. Genetic variants, such as myostatin mutations in Texel sheep and Belgian Blue cattle, enhance muscle mass but may compromise reproductive efficiency. Nutritional interventions, including the plane of nutrition, supplementation strategies, and environmental stressors such as heat and stocking density, significantly influence muscle fiber composition and carcass traits. This review provides a comprehensive overview of skeletal muscle programming in ruminants, tracing the developmental trajectory from progenitor cell differentiation to postnatal growth and maturation. These insights underscore the need for integrated approaches combining maternal diet optimization, molecular breeding, and precision livestock management to enhance muscle growth, meat quality, and production sustainability in ruminant systems.

## Simple Summary

Muscle development in livestock plays a major role in determining meat quality and production efficiency. This study investigated the impact that nutrition before and after birth, genetics, and environmental conditions have on muscle growth in cattle and sheep. The effects of poor or excessive nutrition in pregnant animals on the muscle structure and development of their offspring, which can impact their lifetime performance, are explained in this study. We also describe how muscle grows after birth through the action of special cells, called satellite cells, which help build and repair muscle. This study further discusses how genes and hormones, such as the growth hormone and insulin-like growth factor, regulate these processes. Certain breeds are naturally more muscular due to specific gene mutations, although these can also cause challenges, such as difficult births. Feeding strategies and proper animal care were also shown to affect muscle growth and meat quality. Our findings suggest that improving feeding during pregnancy, selecting the right breeds, and managing postnatal care can all help farmers produce better-quality meat while supporting animal health and farm sustainability. This review will help the livestock industry make informed decisions that benefit both producers and consumers in terms of muscle and meat production.

## 1. Introduction

Muscle growth and development are closely associated with overall meat production in ruminants, with the lean-to-fat ratio serving as an indicator of production efficiency. However, meat quality is primarily determined by muscle tenderness and marbling. As all muscle fibers are formed before birth [1,2], manipulating prenatal muscle development offers a strategic approach to managing overall beef production [3]. Both prenatal and postnatal phases of muscle development are critical for effective myogenesis and intramuscular fat deposition (marbling) [4,5]. Muscles are the organs of lower priority in nutrient partitioning; their development begins during early gestation and continues across gestation [6,7]. Limited nutrition during pregnancy can affect overall muscle development, leading to a lifelong effect on meat quality and production [8,9,10].

Both prenatal and postnatal phases of muscle development are essential for myogenesis and the marbling effect. Prenatal muscle development involves the hyperplasia of myogenic cells, while postnatal growth is mainly driven by hypertrophy, which begins in late gestation and continues throughout puberty [6,7,11]. Dermomyotome progenitor cells (dPC), which give rise to the dermatome and myotome [12,13], are regulated by myogenic regulatory factors and differentiate into myogenic cells [14,15,16], forming primary myofibers during embryogenesis. Non-myogenic cells, primarily from the dermatome region of the dermomyotome, differentiate into fibro-adipogenic progenitors under the influence of key transcription factors [17]. The maternal nutritional implications during gestation and neonatally were found to impact both myogenic and adipogenic cells postnatally, either in terms of hyperplasia or hypertrophy [18,19].

Skeletal muscle constitutes the main component of meat, making its growth and development essential in the livestock industry. While prenatal muscle development establishes the number of muscle fibers, postnatal muscle growth is largely hypertrophic, relying on satellite cell proliferation, protein accretion, and hormonal regulations [20,21,22,23]. Satellite cells, the resident muscle stem cells, play a pivotal role in postnatal muscle hypertrophy by contributing additional nuclei to growing muscle fibers, thereby supporting protein synthesis and fiber enlargement [24]. The activity of these cells is modulated by nutritional status, hormonal signals such as the growth hormone and insulin-like growth factor-1, and environmental factors [5,24,25]. Adequate nutrition, particularly during gestation, enhances satellite cell proliferation and differentiation, promoting muscle fiber hypertrophy and improving meat yield and quality [23,24]. Conversely, nutrient restriction during critical postnatal windows can impair satellite cell function, limit muscle growth potential, and negatively affect meat characteristics such as tenderness and marbling. Moreover, the balance between muscle fiber growth and intramuscular fat deposition is modulated by interactions between satellite cells and fibro-adipogenic progenitors, both of which respond to nutritional and hormonal signals [4,5,21,23]. Understanding these complex regulatory mechanisms is essential for developing feeding and management strategies that optimize muscle development and marbling, thereby enhancing both the quantity and quality of ruminant meat production.

Ruminants exhibit variations in muscle development due to genetic, nutritional, and environmental factors. Understanding the mechanism underlying postnatal muscle growth can aid in optimizing production practices to ultimately improve meat yield and quality, while addressing the challenges related to sustainability and feed efficiency. This review provides a comprehensive overview of skeletal muscle programming in ruminants, tracing the developmental trajectory from progenitor cell differentiation to postnatal growth and maturation. We will critically evaluate the impact of maternal nutrition on fetal myogenesis, elucidating the downstream consequences for beef production traits and meat quality attributes.

## 2. Prenatal Muscle Development in Ruminants

### 2.1. Fetal Programming and Myogenesis

The fetal programming hypothesis, first proposed by Barker and colleagues, suggests that insults or adverse environmental conditions during gestation, such as maternal undernutrition, induce persistent structural and functional adaptations in fetal tissues [26]. Epidemiological and experimental studies in ruminants and humans demonstrate the fact that nutritional stress during critical developmental windows (e.g., myogenesis and adipogenesis) disrupts organogenesis through gene expression regulation and altered maternal–fetal resource partitioning [3,6,7,18,19].

Muscle development is a highly regulated process. It begins during embryogenesis and continues through fetal and postnatal stages [6,7,11]. Muscle fibers, or myofibers, are the fundamental structural units of skeletal muscle, formed exclusively during prenatal development in livestock species [1,2]. Understanding the mechanisms underlying myogenesis during this critical period is essential, as events occurring at this stage profoundly impact postnatal growth, muscle composition, and meat quality [27].

**Figure 1:**
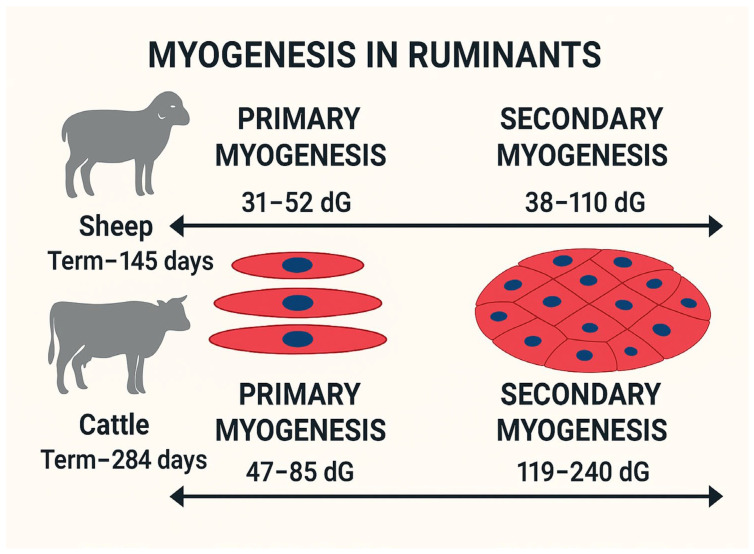
Timeline indicating myogenesis in ruminant models based on days of gestation (dG) [7,18,31,32].

Prenatal myogenesis can be divided into primary and secondary myogenesis. Primary myogenesis occurs during the embryonic stage, forming primary muscle fibers that serve as templates for secondary fibers [16]. Secondary myogenesis, during the fetal stage, generates most muscle fibers from a pool of dPC [28]. These dPCs during embryogenesis initiate primary myogenesis by expressing myogenic regulatory factors (MRFs), including myogenic factor 5 (*MYF5*), *MYOD*, *MYOG*, and *MRF4*; this converts dPC into differentiated muscle fibers [29,30]. Although these primary muscle fibers are limited in number, their role as templates for subsequent fiber formation highlights their importance for determining the overall muscle structure. The primary myogenesis in ruminants varies by days of gestation (dG) from 31 to 52 to 47–85 for sheep and cattle, respectively; see Figure 1 [7,31].

Secondary myogenesis occurs during the fetal stage of development, accounting for most of the fibers formed in farm animals; see Figure 1. Secondary myogenesis in ruminants varies by timing: mid-gestation in cattle (ranging 119–240 days of gestation; term ~284 days) [7,18], and early-to-mid-gestation in sheep (ranging 38–110 days of gestation; term ~145 days) [31,32]. Secondary muscle fibers arise from the fusion of proliferating myogenic cells, and increasing the number of myogenic dPCs, which result in more fiber formation. The number of muscle fibers formed during the fetal stage is directly influenced by the availability and proliferation of myogenic dPC, and it is highly sensitive to nutrient availability [2].

Considering the timeline of muscle development in ruminants (see Figure 1), the early-to-mid-gestation period is critical in ruminants, and limited maternal nutrition can have life-long effects by reducing the number of myogenic dPCs.

### 2.2. Maternal Nutrition and Muscle Development

Muscles are the organs of lower priority for nutrient partitioning during fetal programming [6]. Most studies on maternal nutrition and developmental muscle programming in ruminants have highlighted the lasting effects of both maternal restriction and overnutrition [9,33,34,35,36,37,38,39,40,41,42]. Alterations in maternal nutrition were investigated to determine their impact on overall muscle development through gene expression regulation. The restricted or limited nutrition research in ruminant models is focused on studying the consequences of escalating feed input costs, which account for up to 70% of total expenditures in livestock production [43]. The rise in feed costs is attributed to supply chain vulnerabilities and extreme weather conditions, posing significant challenges for cattle farmers and ranchers [44]. However, overnutrition models in ruminants have been studied to enhance marbling and myogenesis during skeletal muscle development both prenatally and neonatally [33,34,35,45]. The impact of prenatal maternal nutrition on myogenesis in ruminants has been explained extensively in the literature [3,5,6,11,16,45]. However, this review will focus on the genomic aspects of prenatal myogenesis, which is the least explored area in ruminant models.

In terms of maternal nutrient restriction, different studies have highlighted the impacts on fetal myogenesis due to alterations in fetal gene expression profiles [9,33,34,36,37,38,40,41,42,46]. During the periconceptual period, ranging 18 days before and 6 days after ovulation, the ewes received restricted nutrition (half the maintenance requirements) and showed a decrease in muscle fiber count compared to those that received 1.5 times the maintenance requirements [38]. In the periconceptual cattle model, the maternal nutrient restriction (Average Daily Gain, ADG, −0.08 kg/d) during the first 50 dG altered the expression of MRFs, *MYOG*, and *MYOD1*, in fetal hind limb muscle tissues compared to fetuses from heifers provided with 0.5 kg/d ADG [41]. In the sheep model, from early gestation to parturition (45–135 dG, term ~147 days), when both under- and over-nutrition models were studied (under, ADG = 0.047 kg/d; over, ADG = 0.226 kg/d; control, ADG = 0.135 kg/d), the increasing trend in the overall cross-sectional area of fetal semitendinosus and longissimus muscles fibers was observed from 90 dG to parturition, which is inconsistent with fetal growth trajectory, which showed the downregulation of *MYF5* and *PAX7* [36]. These transcriptomic changes were found to be negatively associated with the myogenesis of developing lambs at birth, as evidenced by alterations in their metabolite profiles [34]. In the cattle model of maternal nutrient restriction (ADG = 0.59 kg/d), from 147 to 247 dG for mid-to-late gestation, the fetuses showed an increase in the expression of *MYOD1* and *MYOG* in longissimus muscle compared to fetuses from cows receiving 1.11 kg/d ADG [9]. All these studies from ruminant models highlight how maternal nutrient restriction across gestation impacts the expression profiles of MRF, leaving lasting impacts on overall fetal myogenesis and offspring metabolic profiles at birth.

Like maternal nutrient restriction, overnutrition also led to perturbations in fetal myogenesis and marbling in ruminant models [33,34,35]. However, marbling effects have not been studied extensively in ruminant models, and most research is focused on hepatic and muscle models of fetal development in sheep [33,36,37,47,48,49], with a limited number of studies focusing on beef cattle [9,35]. In the sheep model, where ewes received maternal overnutrition, 150% of maintenance, from 60 days before gestation to parturition, showed lower leptin concentrations neonatally when compared to those that received maintenance requirements only, indicating the potential impacts on lamb marbling and myogenesis [50]. Similarly, in another study with the same nutritional sheep model during the periconceptual period, from 60 days before gestation to 75 dG, MRFs (*MYOD* and *MYOG*) were downregulated in the fetal semitendinosus skeletal muscle tissues of overnutrition ewes compared to fetuses from control ewes [39]. Similarly, as discussed in restricted nutritional models, the ewes received over-nutrition (under, ADG = 0.047 kg/d; over, ADG = 0.226 kg/d; control, ADG = 0.135 kg/d) from early gestation to parturition (45–135 dG, term ~147 days), and also showed downregulation of *MYF5* and *PAX7* [36]. In the cattle model of overnutrition (ADG = 1.11 kg/d) from 147 to 247 dG, mid-to-late gestation, the fetuses showed a downregulation in the expression of *MYOD1* and *MYOG* in longissimus muscle compared to fetuses from cows fed to achieve an ADG of 0.59 kg/d, in contrast to the upregulation observed in the nutrient restriction model [9].

## 3. Postnatal Muscle Development in Ruminants

### 3.1. Mechanisms of Postnatal Muscle Growth

Postnatal muscle growth in ruminants is predominantly a result of muscle fiber hypertrophy, where existing muscle fibers increase in size rather than number. Satellite cells, which are muscle-resident stem cells, play a crucial role in this process by contributing additional nuclei to muscle fibers, thereby enhancing their protein synthesis capacity [5,24,25]. These cells remain quiescent until activated by MRF, *PAX7*, and *PAX3* [51]; once activated, satellite cells proliferate, differentiate, and fuse with existing myofibers, supporting continued muscle growth and repair.

*MYOD* and *MYF5* are the two major MRFs involved in differentiating satellite cells for myogenic proliferation [52,53]. The regulation of *MYF5* activation postnatally is intricately linked to the arginine methylation of *PAX7* by the enzyme CARM1, which catalyzes the recruitment of the Wdr5-Ash2L-MLL2 histone methyltransferase complex to the *MYF5* locus, thereby promoting chromatin remodeling conducive to gene transcription [54,55]. The *MYF5* transcripts are present even in quiescent satellite cells but are sequestered in mRNP granules through miR-31-mediated mechanisms, preventing premature translation. Upon satellite cell activation, these granules disassemble, facilitating the rapid onset of *MYF5* translation and subsequent myogenic activation [20]. Myotubule formation under the influence of *MYOD* induces *MYOG* expression, resulting in the downregulation of *MYF5* [56]. This coordinated activity of *MYOD* and *MYOG* leads to the expression of *MRF4*, regulating terminal differentiation and fusion with muscle fibers [5]. Differences in MRF expression levels among ruminant species and breeds can contribute to variations in muscle growth potential and efficiency, influencing overall meat production.

Hormones play a pivotal role in regulating postnatal muscle development. The IGF-1/mTOR signaling pathway is a key driver of muscle protein synthesis, responding to anabolic stimuli, such as growth hormones (GHs) and nutrient availability [21,22]. The pituitary gland secretes GH and stimulates hepatic IFG-1 production, which subsequently enhances muscle growth by activating satellite cells and promoting protein accretion [21,22]. Additionally, thyroid hormones, insulin, and androgenic steroids contribute to muscle growth by modulating metabolic and anabolic processes in muscle tissue.

Genetic differences among breeds can influence muscle growth potential by influencing gene expression regulation. Myostatin, a negative regulator of muscle growth, plays a significant role in controlling muscle mass [57,58]. Mutations in the myostatin gene result in increased muscle fiber hypertrophy, as observed in Belgian Blue cattle and Texel sheep, leading to the “double muscling” phenotype [59]. While these genetic traits enhance muscle yield, they can also present calving difficulties, reduced fertility, and altered meat texture.

Different ruminant breeds exhibit muscle fiber composition variations, ultimately impacting meat quality. Slow-twitch oxidative (Type I) fibers contribute to endurance and a higher myoglobin content, producing darker, more flavorful meat. Whereas fast twitch glycolytic (Type II) fibers support rapid growth and increased carcass weight, they can reduce meat tenderness [5]. Dairy cattle breeds, in contrast to beef cattle, seem to have higher oxidative metabolism and greater oxidase activities [60]. The Piemontese cattle breed is found to have higher glycolytic fibers than oxidative ones when compared to Belgian blue breed bulls [61]. Similarly, in sheep breeds, the Scottish Blackface breed presented type I muscle fibers compared to the Texel breed, which had type II muscle fibers [62]. Selective breeding programs target optimal fiber type proportions and other growth traits to balance meat quality and production efficiency, which also impact the structure and quality of muscle and meat production in ruminants.

### 3.2. Nutritional and Environmental Influences on Muscle Growth

Nutritional management plays a crucial role in optimizing postnatal muscle growth. Adequate protein intake provides the essential amino acids necessary for muscle protein synthesis, while energy availability influences the efficiency of protein accretion [23]. High-energy diets can enhance muscle growth, but may also increase fat deposition, affecting carcass composition and meat quality [63,64]. In contrast, restricted nutrition during early development can lead to long-term reductions in muscle growth potential [23]. Restricting energy intake to 65% of NRC recommendations in heifers and 50% in cows for 100 days prepartum was found to reduce calf birth and weaning weights and increase calf mortality, thereby impacting herd productivity [65]. In another study, cows were managed, either to maintain a body condition score (BCS) of 5.0 or lose one BCS via energy restriction during mid-gestation, followed by re-alimentation during late gestation [66]. Although birth weights were unaffected, the calves born to restricted cows were lighter from the receiving phase until day 57 post-weaning [66]. However, when carcasses were harvested and evaluated on day 208 after birth, a higher marbling-to-backfat ratio in calves from restricted heifers was found, although the ribeye area, hot carcass weight, meat color, and tenderness were not different [67]. These results were further supported by another study on the same cattle model, where improved marbling-to-backfat ratios and greater tenderness in steaks were found in calves born from restricted dams [68]. In a sheep model, the lambs born to ewes, subjected to nutrient restriction (50% of NRC requirements) from early-to-mid-gestation (day 28 to 78 of gestation) were found to be heavier and to have more backfat when slaughtered on day 280 of age compared to lambs from control-fed ewes (100% of NRC requirements) [69]. Together, these studies emphasize that maternal nutrient intake during mid-gestation programs has progeny outcomes and holds potential for optimizing carcass characteristics, production efficiency, and resilience in overall ruminant production systems; see Figure 1 and Table 1. Future research should aim to integrate the gene expression profiling of satellite cells in offspring with maternal diet records to identify key nutritional signals that modify the myogenic epigenome during fetal life.

Other environmental factors, such as temperature stress, stocking density, and handling practices, can also impact muscle growth in ruminants. Chronic stress leads to elevated cortisol levels, which can promote muscle protein degradation and inhibit growth [70]. Proper livestock management, including adequate space, controlled feeding regimens, and minimized stress during transport and harvest, is essential for maintaining optimal growth rates and meat quality. These environmental factors, along with genetic selection and precise nutritional management, can optimize muscle development, production efficiency, and the consumer acceptance of ruminant meat products.

## 4. Epigenetic Modulations of Muscle Development: From Fetal Programming to Postnatal Outcomes

Skeletal muscle development in ruminants is governed by finely coordinated gene expression programs. Although the foundational MRFs, such as *MYF5*, *MYOD1*, *PAX7*, and *MYOG*, play a central role in driving myogenesis as explained earlier, emerging research highlights the critical influence of epigenetic modulation as a dynamic interface that shapes muscle development. Epigenetics is defined as a heritable change in gene expression, without any change in DNA sequence [71,72]. These changes might persist for multiple cell divisions or generations [73]. This epigenetic regulation, encompassing DNA methylation, histone modifications, and non-coding RNAs, integrates environmental signals, including maternal nutrition and stress, with genetic programs to modulate gene expression patterns [74,75,76]. Importantly, these epigenetic mechanisms act not only during fetal programming but continue to influence satellite cell function and muscle hypertrophy in the postnatal period, ultimately shaping lifelong muscle growth potential, metabolic function, and production efficiency in ruminants [16,77].

During fetal development, the foundation of muscle fiber number and type is laid, a process tightly linked to the activity of MRFs like *MYOD1*, *MYOG*, *PAX7*, and *MYF5* [9,36,39,41]. However, these transcription factors are themselves regulated by epigenetic enzymes such as *DNMT3A*, *EZH2*, and *HDACs*, which modify chromatin accessibility [16]. As it has been shown that maternal methyl-donor supplementation, e.g., methionine supplementation, remodels the fetal methylome in bovine muscle, altering isoform expression and exon usage through DNA methylation changes, the influence of critical myogenic genes like *MYF5* [78,79,80]. Enzymes such as *EZH2* and *HDACs* modulate chromatin accessibility at promoter regions of key myogenic regulators (e.g., *MYOD1* and *MYOG*), affecting muscle fiber differentiation, particularly under conditions of maternal nutrient excess or restriction [81,82]. MicroRNAs also play a pivotal role in the regulation of fetal myogenesis. Maternal obesity has been shown to alter miRNA expression profiles in fetal skeletal muscle, with miRNA *let-7g* identified as a key regulator due to its capacity to suppress stem cell proliferation and promote adipogenic differentiation, thereby influencing muscle development outcomes, as reported in the sheep model [46]. It has also been reported in the mouse model that maternal obesity leads to the increased expression of the early adipogenic marker *Zfp423* in fetal tissues, driven by the hypomethylation of the *Zfp423* promoter region [83]. Collectively, these findings highlight that fetal muscle development in ruminants is governed by tightly integrated epigenetic transcriptional and microRNA-mediated mechanisms, with the maternal environment critically modulating myogenic programming, leaving lifelong impacts on muscle development.

Following birth, further skeletal muscle growth in ruminants is primarily determined by the hypertrophic expansion of established muscle fibers [18,19], which is a process tightly regulated by the activation and differentiation of satellite cells, the resident muscle stem cells [24]. The proliferation and differentiation of muscle fibers are also modulated by epigenetic mechanisms, including histone modifications and microRNAs. It has been reported in the sow model that histone 3 lysine 4 trimethylation (H3K4me3) and histone 3 lysine 9 acetylation (H3K9Ac) in the promoter region of myostatin enhance myostatin expression in the skeletal muscle of offspring from maternal-fed low-protein diets during pregnancy and lactation [84]. In terms of microRNAs, *miR-206* and *miR-1* have also been found to be associated with the inhibition of myostatin expression, which is likely to cause muscular hypertrophy in Texel sheep [85]. It has also been identified that *miR-21* and *miR-103*, as pro-adipogenic markers, are upregulated while *miR-34a*, the anti-adipogenic marker, is downregulated in calf longissimus dorsi muscle from cows receiving the medium plane of nutrition [86]. These studies highlight the role of epigenetic modulations on muscle development from fetal origin to postnatal life. However, current research is still insufficient to fully elucidate the precise impact of these epigenetic factors on classic MRFs such as *MYOD1*, *MYOG*, *PAX7*, and *MYF5*, which are fundamental to myogenic growth and development in ruminants.

## 5. Conclusions

Muscle development in ruminants is a complex, multifactorial process beginning in utero and extending postnatally, intricately regulated by maternal nutrition, genetic programming, satellite cell activity, and environmental conditions. During gestation, especially early-to-mid-gestational stages, maternal nutrition exerts profound effects on fetal myogenesis by altering the expression of MRFs such as *MYOD*, *MYF5*, *MYOG*, and *MRF4*, and key transcription factors like *PAX3* and *PAX7*. These molecular changes affect the number of muscle fibers, the composition of fiber types, and the myogenic potential of satellite cells, with long-term implications for offspring muscle development, carcass traits, and overall production efficiency. Postnatally, muscle growth predominantly occurs through hypertrophy, facilitated by satellite cells that contribute additional nuclei to existing fibers. This process is tightly regulated by the same core MRF network and further modulated by epigenetic factors (e.g., CARM1-mediated chromatin remodeling), nutrient availability, and endocrine signals, such as the growth hormone (GH), insulin-like growth factor-1 (IGF-1), and thyroid hormones. Additionally, genetic factors, including myostatin mutations and breed-specific muscle fiber distributions, play critical roles in determining muscle growth potential, meat quality, and productivity. Evidence suggests that targeted maternal nutritional interventions during gestation can “program” satellite cell activity and muscle characteristics in offspring, highlighting the importance of developmental plasticity in livestock systems. To meet the increasing global demand for high-quality, sustainable meat, future research must adopt a systems biology approach that integrates the genomic and epigenomic profiling of fetal and postnatal muscle tissues with precision nutrition, stress mitigation, and breed-specific management. While substantial progress has been made in understanding muscle programming, its practical implementation in ruminants remains constrained by a reliance on non-ruminant models, undefined parameters for maternal dietary interventions, and inconsistent phenotypic outcomes under diverse production environments. Additionally, the multifaceted nature of epigenetic regulation, along with limited knowledge of its transgenerational heritage, poses further challenges. Advancing the field requires integrated interdisciplinary research efforts towards the development of cost-effective tools to translate molecular insights into scalable on-farm applications that can improve productivity and sustainability in ruminant production systems.

## Figures and Tables

**Table 1:** Studies demonstrating the maternal nutritional effects on pre- and postnatal skeletal muscle development in ruminant models.

Model Animal	Treatments	Gestation Stage	Effects	Reference
Sheep	Maternal nutrient restriction (half maintenance requirements vs. 1.5 times maintenance requirements)	Periconceptual period (18 days before and 6 days after ovulation)	A decrease in muscle fiber count in the restricted nutrition group compared to the control.	[38]
Cattle	Maternal nutrient restriction (ADG −0.08 kg/d vs. ADG 0.5 kg/d)	Early gestation (first 50 days of gestation)	Altered expressions of MRF, *MYOG*, and *MYOD1* in fetal hind limb muscle tissues in the restricted nutrition group.	[41]
Sheep	Maternal nutrient restriction and overnutrition (under: ADG = 0.047 kg/d; over: ADG = 0.226 kg/d; control: ADG = 0.135 kg/d)	Early gestation to parturition (45–135 dG)	Increased cross-sectional area of muscle fibers consistent with fetal growth trajectory; the downregulation of *MYF5* and *PAX7* associated with negative impacts on myogenesis at birth.	[36]
Cattle	Maternal nutrient restriction and overnutrition (ADG = 0.59 kg/d vs. ADG = 1.11 kg/d)	Mid-to-late gestation (147–247 dG)	The increased expression of *MYOD1* and *MYOG* in the restricted nutrition group compared to the overnutrition group.	[9]
Sheep	Maternal overnutrition (150% of maintenance requirements vs. maintenance requirements only)	From 60 days before gestation to parturition	Lower leptin concentration neonatally in the overnutrition group, indicating potential impacts on marbling and myogenesis.	[50]
Sheep	Maternal overnutrition (150% of maintenance requirements vs. control)	Periconceptual period (60 days before gestation to 75 dG)	The downregulation of MRF (*MYOD* and *MYOG*) in the overnutrition group compared to the control group.	[39]
Cattle	Maternal nutrient restriction leads to the loss of body condition score by 1 or maintains a score of 5.0	Mid-gestational nutrient restriction (from start to end of mid-gestation) to study postnatal birth and weaning effects	Early growth suppressed; improved marbling-to-backfat ratio and tenderness in the restricted group.	[66,67,68]
Sheep	Early to mid-gestation maternal nutrient restriction (50% of NRC requirement) vs. control-fed ewes (100% of NRC requirement)	Study the impacts of mid-gestational maternal nutrient restriction on carcass and morphometric measures of lambs at harvesting (day 280 postnatally)	Lambs from restricted ewes were heavier and had more backfat at slaughter.	[69]
Cattle	Prepartum nutrient restriction (heifers 65%, cows 50%; NRC compared to 100%; NRC as control for 100 days)	Postnatal (birth to weaning)	Lower calf birth/weaning weights; increased mortality rates.	[65]
